# Estimation of the Genetic Diversity in Tetraploid Alfalfa Populations Based on RAPD Markers for Breeding Purposes

**DOI:** 10.3390/ijms12085449

**Published:** 2011-08-24

**Authors:** Nevena Nagl, Ksenija Taski-Ajdukovic, Goran Barac, Aleksandar Baburski, Ivana Seccareccia, Dragan Milic, Slobodan Katic

**Affiliations:** 1 Institute of Field and Vegetable Crops, Maksima Gorkog 30, Novi Sad 21000, Serbia; E-Mails: nevena.nagl@ifvcns.ns.ac.rs (N.N.); dragan.milic@ifvcns.ns.ac.rs (D.M.); slobodan.katic@ifvcns.ns.ac.rs (S.K.); 2 Department of Fruit Growing, Viticulture, Horticulture and Landscape Architecture, Faculty of Agriculture, University of Novi Sad, Trg Dositeja Obradovica 8, Novi Sad 21000, Serbia; E-Mail: goranb@polj.uns.ac.rs; 3 Department of Biology and Ecology, Faculty of Natural Sciences, University of Novi Sad, Trg Dositeja Obradovica 2, Novi Sad 21000, Serbia; E-Mails: aleksandar_baburski@yahoo.com (A.B.); ivana_seccareccia@yahoo.com (I.S.)

**Keywords:** alfalfa, breeding, cluster analysis, genetic diversity, RAPD

## Abstract

Alfalfa is an autotetraploid, allogamous and heterozygous forage legume, whose varieties are synthetic populations. Due to the complex nature of the species, information about genetic diversity of germplasm used in any alfalfa breeding program is most beneficial. The genetic diversity of five alfalfa varieties, involved in progeny tests at Institute of Field and Vegetable Crops, was characterized based on RAPD markers. A total of 60 primers were screened, out of which 17 were selected for the analysis of genetic diversity. A total of 156 polymorphic bands were generated, with 10.6 bands per primer. Number and percentage of polymorphic loci, effective number of alleles, expected heterozygosity and Shannon’s information index were used to estimate genetic variation. Variety Zuzana had the highest values for all tested parameters, exhibiting the highest level of variation, whereas variety RSI 20 exhibited the lowest. Analysis of molecular variance (AMOVA) showed that 88.39% of the total genetic variation was attributed to intra-varietal variance. The cluster analysis for individual samples and varieties revealed differences in their population structures: variety Zuzana showed a very high level of genetic variation, Banat and Ghareh were divided in subpopulations, while Pecy and RSI 20 were relatively uniform. Ways of exploiting the investigated germplasm in the breeding programs are suggested in this paper, depending on their population structure and diversity. The RAPD analysis shows potential to be applied in analysis of parental populations in semi-hybrid alfalfa breeding program in both, development of new homogenous germplasm, and identification of promising, complementary germplasm.

## 1. Introduction

Alfalfa (*Medicago sativa* L.) is the most important forage crop with high biomass yield, whose excellent nutritive value makes it ideal for dairy and livestock production [1]. Breeding of alfalfa is rather complex due to its genetic structure. Cultivated alfalfa is a tetraploid (2*n* = 4*x* = 32), perennial, open pollinated legume with polysomic inheritance [2,3]. Since severe inbreeding depression hinders development of inbred lines, all commercial cultivars are synthetic populations generated by crossing different numbers of selected genotypes [4]. Because of the tetraploid structure of the alfalfa genome, cross pollination and severe inbreeding depression, cultivars can exhibit different levels of genetic variation [5]. Therefore, information about germplasm diversity and relationships within and among elite breeding material is of great importance for any efficient and successful alfalfa breeding program. Recent studies support idea of the semi-hybrid breeding of this crop [1]. The concept involves: breeding alfalfas within the population, identification of heterotic germplasm, and the production of seed of the population hybrid [6].

Characterization of genetic variation in alfalfa by using morphological traits is sometimes insufficient, especially when closely related populations or those with narrow genetic base are used [7]. Unlike phenotypic markers, molecular markers detect diversity and differences among and within cultivars directly at DNA level, independently of environmental factors. Different types of molecular markers have been used in alfalfa populations [8–11] and other *Medicago* germplasm sources [12] for estimation of their relationships, variety and ecotype identification [13,14], analysis of population genetic structure [15], and construction of genetic linkage maps [5,16,17]. Markers that are very often used are random amplified polymorphic DNAs (RAPD) [18], which, despite dominance and low reproducibility, allow an inexpensive and rapid analysis of the polymorphisms in many individuals with good coverage of the entire genome [19].

The aim of this research was to determine genetic diversity of five varieties, considered as potential parental populations in semy-hybrid alfalfa breeding program at Institute of Field and Vegetable Crops (Novi Sad, Serbia), and estimate their genetic relationships through RAPD markers. We will also discuss how understanding the genetic variation and population structure of the analyzed breeding material affects their application in alfalfa breeding.

## 2. Experimental Section

### 2.1. Plant Material and DNA Isolation

Five tetraploid alfalfa varieties of different geographic origin, involved in progeny tests were chosen for this study (Table 1) [20].

From each variety, 10 individual samples were taken for DNA isolation and further RAPD analysis. Total genomic DNA was isolated from leaves according to the protocol of Somma [21].

### 2.2. RAPD Analysis

In order to test amplification profiles for polymorphism, readability and reproducibility, sixty decamer primers from ROTH^®^GmbH kits X, Y and Z were initially tested [22] from which seventeen primers were used for further RAPD analysis (Table 2). PCR was carried out in a 25-μL reaction volume containing 2.5 μL buffer; 0.2 mM of each dNTP; 0.5 μM of primer; 2 units of Taq polymerase (Fermentas) and 30 ng of DNA. Reactions were performed in Tpersonal PCR (Biometra) and Mastercycler ep gradient S (Eppendorf) thermocyclers with amplification profile: denaturation at 94 °C for 4 min, followed by 40 cycles with 94 °C for 2 min, 36 °C for 1 min and 72 °C for 2 min, with final elongation on 72 °C for 10 min. PCR products were separated on 1.2% or 1.7% agarose gels containing 0.005% ethidium bromide and visualized under UV light.

### 2.3. Data Analysis

Each fragment amplified using RAPD primers was treated as binary unit character and scored “0” for absence and “1” for presence. In order to measure informativeness of the markers, the polymorphism information content (PIC) for each primer was calculated [23]. PIC provides an estimate of discriminatory power of a marker by taking into account not only the number of alleles at a locus, but also their relative frequencies. Estimation of genetic variation was carried out by using the POPGENE software package version 1.32 [24] for calculation of the following parameters: number of polymorphic loci and their percentage, effective number of alleles per loci [25], expected heterozygosity [26] based on allelic frequencies and Shannon’s index of phenotypic diversity [27] based on marker frequencies. Calculations of all parameters were done separately for each population, and overall for all fifty samples. For estimation of variance components among and within the tested varieties analysis of molecular variance [28] was performed, using the ARLEQUIN 3.11 software [29].

Jaccard’s coefficient of similarity was calculated and dendograms for varieties and individual samples were constructed by using Unweighted Pair Group Method of Arithmetic Mean (UPGMA), using software package NTSYS-PC Version 2.11 [30]. The same software was used to perform the Mantel test [31] of correlation between the cophenetic values and the Jaccard’s similarity coefficients to ascertain reliability of the obtained clusters. Robustness of the clustering pattern was also tested by bootstrap analysis using Free Tree software [32]. Pearson’s correlation coefficient was calculated using the STATISTICA program version 8 [33].

## 3. Results

**Figure 1.**
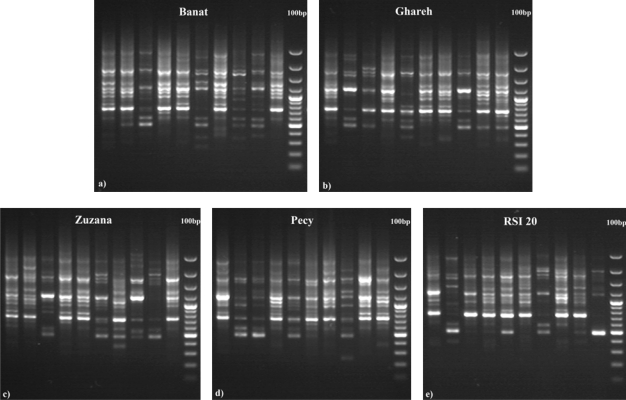
RAPD profiles of 50 individual samples from five alfalfa varieties generated by primer Y15: (**a**) Banat; (**b**) Ghareh; (**c**) Zuzana; (**d**) Pecy; and (**e**) RSI 20, 100 bp GeneRuler^™^ 100 bp DNA ladder plus (Fermentas).

Out of 60 tested RAPD primers, 17 primers generated stable and reproducible bands in all samples during the preliminary primer testing, and later in genetic diversity investigation (Figure 1). A total of 156 polymorphic bands were generated, ranging from 300 to 6000 bp (Table 2), with average number of bands per primer of 10.6 and polymorphism information content (PIC) value of 0.278. The highest number of polymorphic bands was achieved with primer X17 (17 bands), while the most informative primer was Z17 with PIC value of 0.374.

An estimate of genetic variation among and within alfalfa varieties based on RAPD markers is presented in Table 3. Although none of the scored loci was monomorphic in all varieties, they were uniform in some populations, while showing different levels of polymorphism in others. The number and percentage of polymorphic loci, as well as effective number of alleles, were the highest in variety Zuzana. The values of expected heterozygosity in the tested varieties ranged from 0.217 to 0.256, with an average of 0.226 within varieties and overall value of 0.286 among varieties. As a measure of the degree of variation within population, the Shannon’s diversity index was the highest in Zuzana (*I* = 0.375). The mean value within varieties was 0.322 and total genetic diversity across populations was 0.426. Among these five varieties, variety Zuzana exhibited the highest level of variation (*P* = 104, *P*(%) = 66.67, *Ne* = 1.453, *He* = 0.256, *I* = 0.375). A positive Pearson correlation was detected between percentage of polymorphism and Shannon’s diversity index (*r* = 0.995, *p* < 0.001) and between effective number of alleles per locus and expected heterozygosity (*r* = 0.994, *p* < 0.001), suggesting that polymorphism was unevenly distributed among populations. Most of the genetic variability estimated by AMOVA was attributed to variation among individuals within varieties (88.39%) and only 11.61% was found between varieties (Table 4).

**Figure 2.**
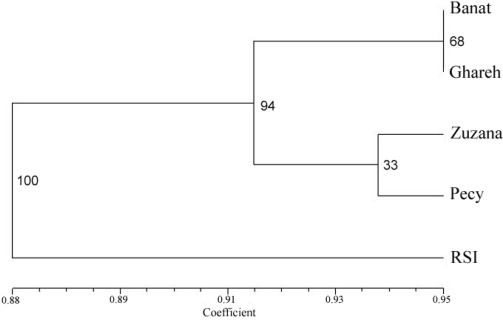
Dendogram of five alfalfa varieties based on unweighted pair group arithmetic mean method cluster analysis of Jaccard’s genetic similarity coefficients. Numbers represent bootstrap confidence limits for 1000 replicates.

UPGMA dendogram was drawn to visualize relationships among five alfalfa varieties (Figure 2). It showed that Banat and Ghareh formed one subcluster with high level of similarity, while Pecy and Zuzana formed another. These four varieties formed a consistent group (94% of bootstraps), while variety RSI 20 was apart (100% of bootstraps). The cophenetic correlation coefficient was high for dendogram clustering populations, with value *r* = 0.885.

**Figure 3.**
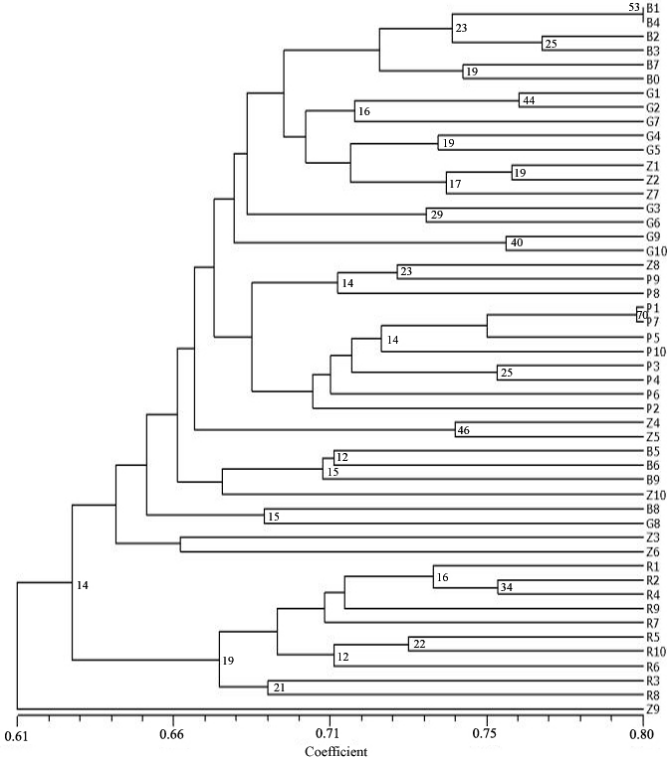
Dendogram of 50 individual samples based on unweighted pair group arithmetic mean method cluster analysis of Jaccard’s similarity coefficient. (B1-B10-Banat, G1-G10-Ghareh, Z1-Z10-Zuzana, P1-P10-Pecy, R1-R10-RSI 20). Numbers represent bootstrap confidence limits for 1000 replicates.

The dendogram representing the relationship between individuals (Figure 3) did not divide all genotypes into distinct groups resembling the analyzed alfalfa varieties. Genotypes from Banat and Ghareh varieties were divided in two groups, while Zuzana was distributed along the dendogram, showing large intrapopulation diversity. The genotypes of variety Pecy formed one cluster with two subclusters: first with eight Pecy genotypes and the second with two Pecy and one Zuzana genotype. RSI 20 genotypes formed distinct cluster. The cophenetic correlation coefficient for dendogram of individuals was *r* = 0.657.

## 4. Discussion

The assessment of genetic diversity and structure of germplasm is essential for the efficient organization of breeding material. With that in mind, the aim of this research was to estimate genetic variation of varieties already included in alfalfa breeding and discuss how that knowledge might affect their involvement in a breeding program.

Since the investigation was done on the valuable breeding material, we estimated genetic diversity by using a larger number of RAPD primers than would usually be used in such investigation [7,8,11], with the aim to generate as many polymorphic bands as possible.

Most alfalfa varieties are genetically broad-based, developed by crossing selected parents and improving their offsprings through several generations. It is well known that decreased heterozygosity and heterogeneity of populations can decrease vigor and productivity. Inbreeding tetraploid alfalfa results in more substantial depression in vigor (yield) than might be expected based solely on the decrease in heterozygosity [1]. The large variation may improve the adaptation of cultivars to a wide range of environments. However, the high level of genetic variation may also slow down genetic progress by slowing the concentration of desirable alleles and limiting the purging of deleterious alleles [1].

Genetic diversity determined in this study was high, which agrees with previous research on tetraploid alfalfa [11,34]. The AMOVA revealed a very high distribution of genetic variation within varieties, which agrees with results of investigations done on a range of diverse alfalfa populations of various origins [8,9,14]. This contrasts with results of [35], where 50% of the total variance was attributed to within-population genetic variability, but landraces and varieties investigated were of distinctly different origins. The large genetic variation at the intrapopulation or variety levels [36] can be explained by alfalfa allogamy, its autotetraploidy and sexual propagation. It can also be a reflection of differences in the amount and type of germplasm used for variety development [7]. It should also be mentioned that, since RAPD markers are dominant, a certain amount of present genetic variation is underestimated.

The dendogram of cluster analysis of individuals clearly illustrated population structure of the tested varieties. It showed the presence of subpopulations in Banat and Ghareh and different levels of similarity between them. It also showed that varieties Pecy and RSI 20 are more uniform, forming tight, clearly defined clusters. As the estimates of genetic diversity indicated, variety Zuzana showed very high level of variation. The obtained results are in accordance with breeding history of the analyzed varieties: Zuzana is old, very adaptable synthetic variety developed by crossing large number of parents, Banat and Ghareh were developed mainly by collecting local germplasm with a certain level of uniformity in yield and morphological traits, while Pecy and RSI 20 resulted from intensive breeding programs.

In our opinion, the use of investigated germplasm in the breeding programs would depend on their population structure and diversity. The populations with high level of genetic variation like Zuzana might be used as a source for selection of desirable germplasm, for development of new varieties. Sub-populations in Banat and Ghareh could be used in development of new, unique and homogenous germplasm. The germplasms with a higher level of uniformity, like Pecy and RSI 20 in this investigation, could be used as potential parents in semy-hybrid breeding program, for producing heterozygous hybrid progenies. The previous research in alfalfa showed that selection of very diverse parental genotypes according to their molecular markers distance has not been successful in predicting heterosis [37–39]. However, the populations selected for our investigation, although with different geographic origin, were bred for growing in similar agroecological conditions. Therefore, we suggest that if highly adapted, relatively uniform populations, with optimal coefficient of similarity, are taken as the parental components, the chances of predicting hybrid effect might increase.

## 5. Conclusions

The presented results confirmed that RAPD analysis can be successfully used in estimation of genetic diversity within and among alfalfa varieties. The obtained data were clear and reproducible, showing that this simple and quick molecular technique is very useful for purposes such as ours. This type of molecular marker analysis might potentially be used in development of new, uniform germplasm and identification of promising, complementary germplasm aiming at reduced number of necessary crossings and therefore making future alfalfa breeding programs more efficient.

## Figures and Tables

**Table 1.** Description of alfalfa varieties.

**Variety**	**Description**
Banat (NS Banat ZMS II)	An old variety developed at the Institute of Field and Vegetable Crops, Novi Sad, Serbia by individual selection from local populations (Pannonian type of alfalfa). Has rapid initial growth and fast regrowth after cutting. Plant height at early flowering is over 80 cm. Proportion of leaves in green forage yield is 450–500 g kg^−1^. Green forage yield is about 80 t ha^−1^, hay yield is 15–20 t ha^−1^. Resistant to drought, low temperatures and frequent cuttings.
Ghareh (Ghareh Yon Geh)	Variety developed at the Institute Karaj in Iran, the center of alfalfa origin. Well adapted to agroecological conditions of Serbia (resistant to drought and low temperature). Has tall, large plants that regrow fast after cutting (37.1 cm) and high hay yield (16–19 t ha^−1^).
Zuzana	An old variety developed at the breeding station in Zelezice, Brno, Czech Republic, with good adaptation to agroecological conditions of Serbia. Has good dry matter yield (14 t ha^−1^), a larger number of shorter internodes (tolerance to lodging), slower regrowth after cutting (higher dormancy), and higher susceptibility to drought. Represents a transition between Pannonian and Western European type of varieties.
Pecy	An old French variety developed by company R2N. A typical variety of the Western European type. Has good resistance to lodging and main alfalfa diseases. Well able to withstand low temperatures but is susceptible to drought. Has an exceptional quality, with high proportion of leaves in yield (48–56%), and larger number (13) of short internodes (5.2 cm).
RSI 20	Variety originating from Spain. Early-maturing variety with high dry matter yield (17.9 t ha^−1^) and excellent quality (crude protein content of 22.2%). Has low dormancy (fast regrowth after cutting −39.4 cm), tolerance to high temperatures and drought, but it is sensitive to cold. Because of smaller number (10) of long internodes (6.3 cm) it is susceptible to lodging.

**Table 2.** Description of oligonucleotide primers used for random amplified polymorphic DNA (RAPD) analysis.

**Primer**	**Sequence (5′-3′)**	**Max. No. of Bands**	**Band Size Range (bp)**	**PIC**
X07	GAGCGAGGCT	14	400–2000	0.267
X09	GGTCTGGTTG	12	600–3000	0.286
X12	TCGCCAGCCA	13	400–2000	0.174
X17	GACACGGACC	17	400–3000	0.368
Y02	CATCGCCGCA	7	900–3500	0.314
Y05	GGCTGCGACA	11	700–4000	0.285
Y06	AAGGCTCACC	8	1000–3500	0.358
Y07	AGAGCCGTCA	14	700–4000	0.291
Y10	CAAACGTGGG	9	650–4000	0.320
Y11	AGACGATGGG	13	300–2500	0.308
Y13	GGGTCTCGGT	10	500–2800	0.345
Y15	AGTCGCCCTT	9	350–3000	0.361
Z01	TCTGTGCCAA	6	450–1600	0.011
Z07	CCAGGAGGAC	11	600–3000	0.158
Z12	TCAACGGGAC	8	1000–6000	0.242
Z14	TCGGAGGTTC	8	900–3500	0.256
Z17	CCTTCCCACT	10	400–5000	0.374

PIC: polymorphism information content.

**Table 3.** Estimates of genetic variation in alfalfa varieties using RAPD markers.

**Variety**	***P* (No.)**	***P* (%)**	***Ne***	***He***	***I***
Banat	90	57.69	1.389 ± 0.400	0.220 ± 0.212	0.323 ± 0.300
Ghareh	88	56.41	1.387 ± 0.389	0.217 ± 0.209	0.319 ± 0.298
Zuzana	104	66.67	1.453 ± 0.399	0.256 ± 0.208	0.375 ± 0.292
Pecy	88	56.41	1.393 ± 0.407	0.220 ± 0.215	0.322 ± 0.305
RSI 20	87	55.77	1.385 ± 0.402	0.217 ± 0.213	0.318 ± 0.302
Mean	91.4	58.59	1.399 ± 0.399	0.226 ± 0.211	0.322 ± 0.299
Overall	129	82.69	1.498 ± 0.377	0.286 ± 0.189	0.426 ± 0.257

*P* (No): number of polymorphic loci; *P* (%): percentage of polymorphic loci; *Ne*: Effective number of alleles; *He*: expected heterozygosity; *I*: Shannon’s information index.

**Table 4.** Analysis of molecular variance (AMOVA) for five alfalfa varieties.

**Source of Variation**	**Df**	**SSD**	**Variance Components**	**Percentage Variation**	**P**
Among populations	4	143.464	2.03623	11.61	<10^−5^
Within populations	45	697.666	15.5369	88.39	<10^−5^

Total		841.130	17.53992		

Df: degrees of freedom; SSD: sum of squared deviations; P: probability of obtaining a more extreme component estimate by chance alone, estimated by computing 16,000 permutations.
